# Supplementary material for: Dynamics of the Tumor Immune Microenvironment during Neoadjuvant Chemotherapy of High-Grade Serous Ovarian Cancer
Source: Cancers (Basel). 2022 May 6;14(9):2308. doi: 10.3390/cancers14092308 (PMC9104540; doi:10.3390/cancers14092308)
Supplement: Supplementary file 1 [file cancers-14-02308-s001.zip › cancers-1664680-supplementary.pdf]

Table S1. Gene content in the TruSight Tumor 170 panel

Small nucleotide variants and indels (from DNA)									
AKT1	*BRIP1*	*CREBBP*	*FANCI*	*FGFR2*	*JAK3*	*MSH3*	*PALB2*	*RAD51B*	*TET2*
AKT2	*BTK*	*CSF1R*	*FANCL*	*FGFR3*	*KDR*	*MSH6*	*PAX3*	*RAD51C*	*TMPRSS2*
AKT3	*CARD11*	*CTNNB1*	*FBXW7*	*FGFR4*	*KIT*	*MTOR*	*PAX7*	*RAD51D*	*TP53*
ALK	*CCND1*	*DDR2*	*FGF1*	*FLT1*	*KMT2A*	*MUTYH*	*PDGFRA*	*RAD54L*	*TSC1*
APC	*CCND2*	*DNMT3A*	*FGF2*	*FLT3*	*KRAS*	*MYC*	*PDGFRB*	*RB1*	*TSC2*
AR	*CCNE1*	*EGFR*	*FGF3*	*FOXL2*	*MAP2K1*	*MYCL1*	*PIK3CA*	*RET*	*VHL*
ARID1A	*CD79A*	*EP300*	*FGF4*	*GEN1*	*MAP2K2*	*MYCN*	*PIK3CB*	*RICTOR*	*XRCC2*
ATM	*CD79B*	*ERBB2*	*FGF5*	*GNA11*	*MCL1*	*MYD88*	*PIK3CD*	*ROS1*	
ATR	*CDH1*	*ERBB3*	*FGF6*	*GNAQ*	*MDM2*	*NBN*	*PIK3CG*	*RPS6KB1*	
BAP1	*CDK12*	*ERBB4*	*FGF7*	*GNAS*	*MDM4*	*NF1*	*PIK3R1*	*SLX4*	
BARD1	*CDK4*	*ERCC1*	*FGF8*	*HNF1A*	*MET*	*NOTCH1*	*PMS2*	*SMAD4*	
BCL2	*CDK6*	*ERCC2*	*FGF9*	*HRAS*	*MLH1*	*NOTCH2*	*PPP2R2A*	*SMARCB1*	
RBCL6	*CDKN2A*	*ERG*	*FGF10*	*IDH1*	*MLLT3*	*NOTCH3*	*PTCH1*	*SMO*	
BRAF	*CEBPA*	*ESR1*	*FGF14*	*IDH2*	*MPL*	*NPM1*	*PTEN*	*SRC*	
BRCA1	*CHEK1*	*EZH2*	*FGF23*	*INPP4B*	*MRE11A*	*NRAS*	*PTPN11*	*STK11*	
BRCA2	*CHEK2*	*FAM175A*	*FGFR1*	*JAK2*	*MSH2*	*NRG1*	*RAD51*	*TERT*	
Copy number variations (from DNA)									
AKT2	*BRCA2*	*CHEK1*	*ERCC2*	*FGF5*	*FGF14*	*FGFR4*	*MDM4*	*NRG1*	*RAF1*
ALK	*CCND1*	*CHEK2*	*ESR1*	*FGF6*	*FGF19*	*JAK2*	*MET*	*PDGFRA*	*RET*
AR	*CCND3*	*EGFR*	*FGF1*	*FGF7*	*FGF23*	*KIT*	*MYC*	*PDGFRB*	*RICTOR*
ATM	*CCN21*	*ERBB2*	*FGF2*	*FGF8*	*FGFR1*	*KRAS*	*MYCL1*	*PIK3CA*	*RPS6KB1*
BRAF	*CDK4*	*ERBB3*	*FGF3*	*FGF9*	*FGFR2*	*LAMP1*	*MYCN*	*PIK3CB*	*TFRC*
BRCA1	*CDK6*	*ERCC1*	*FGF4*	*FGF10*	*FGFR3*	*MDM2*	*NRAS*	*PTEN*	
Fusions and splice variants (from RNA)									
ABL1	*BRAF*	*EML4*	*ETV4*	*FGFR4*	*KIF5B*	*MYC*	*NTRK3*	*PIK3CA*	*TMPRSS2*
AKT3	*BRCA1*	*ERBB2*	*ETV5*	*FLI1*	*KIT*	*NOTCH1*	*NTRK3*	*PPARG*	
ALK	*FRCA2*	*ERG*	*EWSR1*	*FLT1*	*KMT2A*	*NOTCH2*	*PAX3*	*RAF1*	
AR	*CDK4*	*ESR1*	*FGFR1*	*FLT3*	*MET*	*NOTCH3*	*PAX7*	*RET*	
AXL	*CSF1R*	*ETS1*	*FGFR2*	*JAK2*	*MLLT3*	*NRG1*	*PDGFRA*	*ROS1*	
BCL2	*EGFR*	*ETV1*	*FGFR3*	*KDR*	*MSH2*	*NTRK1*	*PDGFRB*	*RPS6KB1*	

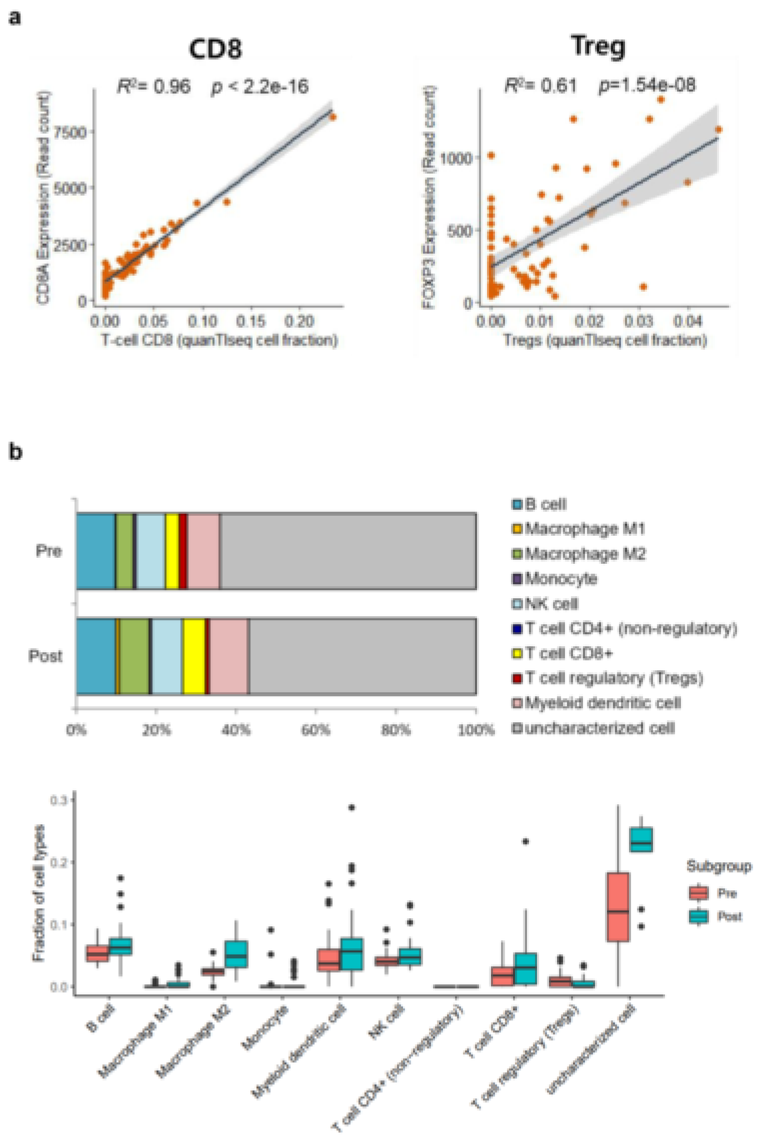

Figure S1: quanTIseq analysis of immune cell fraction (a) Correlation between immune cell frac-tion and maker expression (b) Comparison of immune cell fraction between pre- and post-NAC tumor samples

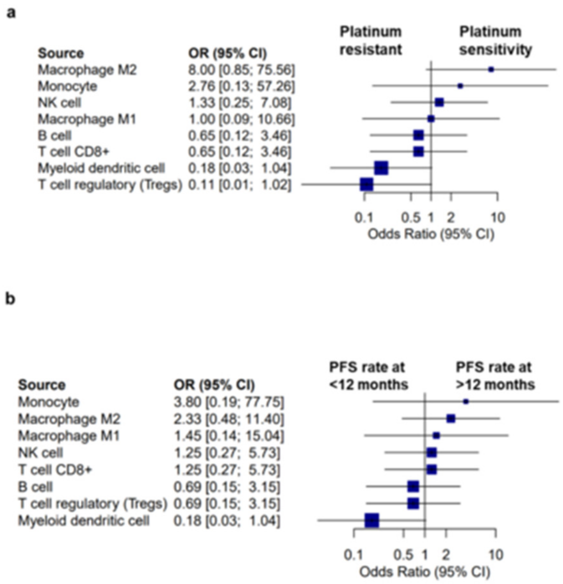

Figure S2: Forest plot showing the association between immune cell fractions, platinum sensitivity, and progression-free survival rate at 12 months

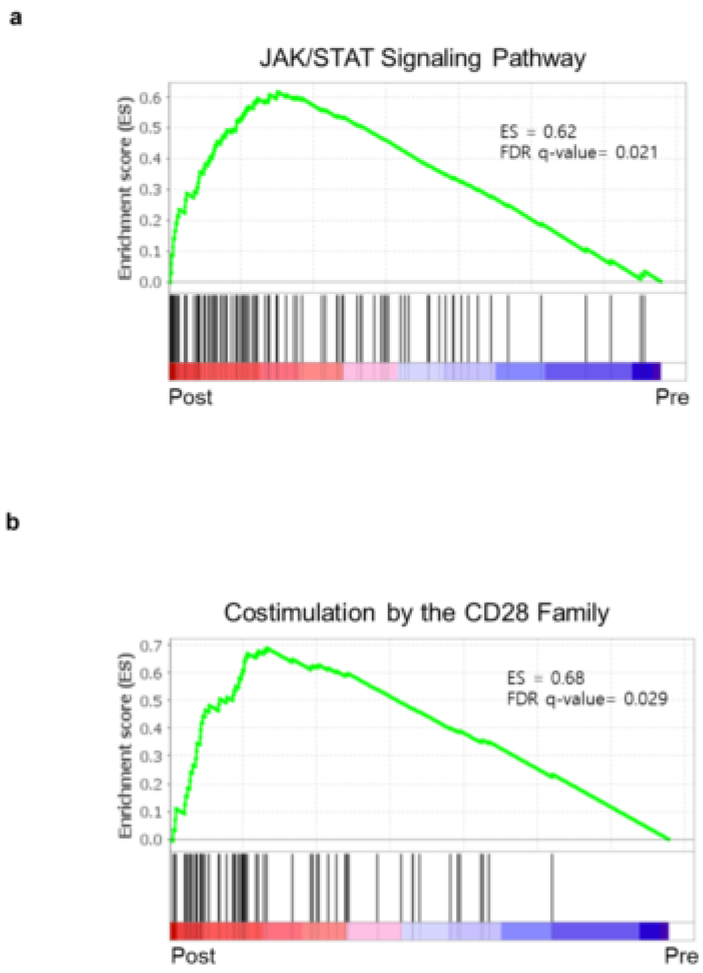

Figure S3: Gene set en-richment analysis of differentially expressed genes in pre- and post-NAC samples
